# Evaluating Molecular Mechanism of Viral Inhibition of Aerosolized Smart Nano-Enabled Antiviral Therapeutic (SNAT) on SARS-CoV-2-Infected Hamsters

**DOI:** 10.3390/toxics12070495

**Published:** 2024-07-05

**Authors:** Anais N. Bauer, John F. Williams, Lok R. Pokhrel, Selena Garcia, Niska Majumdar, Jeffrey B. Eells, Paul P. Cook, Shaw M. Akula

**Affiliations:** 1 Department of Microbiology and Immunology, Brody School of Medicine, East Carolina University, Greenville, NC 27834, USA; bauera22@students.ecu.edu (A.N.B.); williamsjohnf@ecu.edu (J.F.W.); sgarcia9@eagles.nccu.edu (S.G.); majumdarn22@students.ecu.edu (N.M.); 2 Department of Public Health, Brody School of Medicine, East Carolina University, Greenville, NC 27834, USA; 3 Department of Anatomy and Cell Biology, Brody School of Medicine, East Carolina University, Greenville, NC 27834, USA; eellsj17@ecu.edu; 4 Department of Internal Medicine, Brody School of Medicine, East Carolina University, Greenville, NC 27834, USA; cookp@ecu.edu

**Keywords:** nanomedicine, SARS-CoV-2, miRNA, virus, antiviral, S protein

## Abstract

Smart Nano-enabled Antiviral Therapeutic (SNAT) is a promising nanodrug that previously demonstrated efficacy in preclinical studies to alleviate SARS-CoV-2 pathology in hamsters. SNAT comprises taxoid (Tx)-decorated amino (NH_2_)-functionalized near-atomic size positively charged silver nanoparticles (Tx–[NH_2_-AgNPs]). Herein, we aimed to elucidate the molecular mechanism of the viral inhibition and safety of aerosolized SNAT treatment in SARS-CoV-2-infected golden Syrian hamsters. High-resolution transmission electron microscopy (HR-TEM) coupled with energy dispersive spectroscopy (EDS) and ELISAs showed SNAT binds directly to the SARS-CoV-2 virus by interacting with intact spike (S) protein, specifically to S2 subunit. SNAT (≥1 µg/mL) treatment significantly lowered SARS-CoV-2 infections of Calu-3 cells. Extraction-free whole transcriptome assay was used to detect changes in circulatory micronome in hamsters treated intranasally with SNAT (two doses of 10 µg/mL of 2 mL each administered 24 h apart). Uninfected hamsters treated with SNAT had altered circulatory concentrations of 18 microRNAs (8 miRNAs upregulated, 10 downregulated) on day 3 post-treatment compared to uninfected controls. SNAT-induced downregulation of miR-141-3p and miR-200b-3p may reduce viral replication and inflammation by targeting Ythdf2 and Slit2, respectively. Further, SNAT treatment significantly lowered IL-6 expression in infected hamster lungs compared to untreated infected hamsters. Taken together, we demonstrate that SNAT binds directly to SARS-CoV-2 via the S protein to prevent viral entry and propose a model by which SNAT alters the cellular miRNA-directed milieu to promote antiviral cellular processes and neutralize infection. Our results provide insights into the use of low-dose intranasally delivered SNAT in treating SARS-CoV-2 infections in a hamster model.

## 1. Introduction

In the quest to identify effective treatments against COVID-19, researchers have been exploring the potential of nanomedicines, also called nanodrugs. Nanodrugs are drug delivery systems that utilize nanoparticles to enhance the therapeutic properties of drugs, including their stability, bioavailability, and targeted delivery [1,2,3]. Nanodrugs are also being studied as treatments against cancers, microbial infections, and other diseases [4,5,6,7]. In 1995, the anticancer drug called Doxil became the first Food and Drug Administration (FDA)-approved nanodrug on the market. Interest in the field continues to grow, with approximately 100 nanomedicines approved by the FDA and European Medicines Agency (EMA) on the market in 2021 [8,9]. There are many types of nanocarriers for drugs, including liposomes, nanocrystals, and inorganic nanoparticles [10]. The nanoparticle chosen may inherently possess therapeutic properties, be coated with a therapeutic, or have a combination of the two [10].

Nanotechnology has contributed to several advances in antiviral treatments, addressing some of the limitations of current strategies. Compared to traditional antivirals, nanomedicines can have improved in terms of their safety and efficacy, and they serve as an alternate therapy to prevent drug resistance [10,11,12]. Nanoparticles can be modified to carry chemotherapeutic drugs on their surface, some of which have already been used to treat viral infections, enhancing their delivery and effectiveness [13]. Their small size imparts a large surface area to volume ratio, improving their bioavailability and potency [11]. The greatest concern surrounding nanomedicine is potential off-target cytotoxicity. Because small particles <150 nm have the potential to enter cells, there is potential for cytotoxic effects [14]. In addition, the biological and environmental effects of nanoparticle exposure are still under investigation [15]. However, design elements such as surface coatings can be included in the engineering of nanodrugs to limit toxicity and preclinical in vitro/in vivo studies serve as a key step to evaluate cytotoxicity during drug development [16].

Smart Nano-enabled Antiviral Therapeutic, or SNAT, is a novel nanomedicine that has been shown to have antiviral effects against SARS-CoV-2 in our in vivo preclinical study [17]. It comprises positively charged silver nanoparticles (AgNPs) that are taxoid (Tx)-decorated and amino (NH_2_)-functionalized (Tx–[NH_2_-AgNPs]) [17]. The “seed” NH_2_-AgNPs are spherical with a mean TEM diameter of 5.8 nm ± 2.8 nm (S.D.) and are collectively embedded within Tx molecules in solution [17]. This study involved cytotoxicity assays and an infection experiment using male golden Syrian hamsters (*Mesocricetus auratus*). We demonstrated that administration of inhaled SNAT at 2 h and 48 h post-infection with SARS-CoV-2 could significantly reduce virus titer, improve lung health, and counteract virus-induced body weight loss in hamsters [17]. However, the molecular mechanisms underlying SNAT inhibition of SARS-CoV-2 in hamsters were unclear. Therefore, in this study, we designed experiments to uncover molecular mechanisms by which SNAT may alleviate SARS-CoV-2-induced pathology using a combination of virology, next-generation sequencing (NGS), and biophysical and biochemical approaches.

Previous research has indicated that AgNPs can bind to viral surface proteins, potentially interfering with viral entry or hampering the structural integrity of the virus [18]. Evidence also suggests that nanoparticles may impact the cellular environment, such as reducing inflammation or altering the transcriptome [19,20]. Thus, we investigated two plausible mechanisms by which SNAT may interfere with SARS-CoV-2 infection: (i) by binding with the virus to directly hinder viral infection of cells and (ii) by altering the cellular microRNA-directed milieu to alleviate virus-induced inflammation and pathology.

## 2. Materials and Methods

### 2.1. Cells and Virus

Human lung adenocarcinoma-derived Calu-3 and Vero cells (American Type Culture Collection (ATCC), Manassas, VA, USA) were cultured in Dulbecco modified Eagle medium (DMEM) (Invitrogen, Carlsbad, CA, USA) containing 10% charcoal-stripped fetal bovine serum, L-glutamine, and antibiotics as per standard laboratory protocol [17].

All SARS-CoV-2-related work was performed in BSL-3 laboratory. SARS-CoV-2 (isolate USA-WA1/2020; bei RESOURCES, Manassas, VA, USA) was propagated in Vero cells following standard procedures [17]. Upon propagation, the virus was purified, concentrated using a 10–30% sucrose gradient in an ultracentrifuge (Beckman L8-55), and the yield was titrated following standard protocols [17]. The research was approved by the Office of Prospective Health/Biological Safety for the use of biohazardous agent (SARS-CoV-2) with the registration number 20–01 and titled: Host response to COVID-19 infection in Eastern North Carolina. Our Animal Use Protocol, entitled “Effect of SNAT on SARS-CoV-2 infection of hamsters” (AUP #K177), was approved by ECU Institution’s Animal Care and Use Committee (approval date: 11 December 2020). In vitro effects of SNAT on SARS-CoV-2 infection of cells were assessed by viral titration following standard procedures [17].

### 2.2. SNAT Synthesis and Characterization

The details of SNAT (Tx–[NH_2_-AgNPs]) synthesis were previously described by our group [17].

Multimethod complementary techniques were used that allowed for a detailed physicochemical characterization of SNAT. To this end, we used electron microscopy (TEM, Philips EM 420; HR-TEM, Titan 80-300 TEM, CAMCOR, Oregon, Eugene, OR, USA) coupled with an energy dispersive spectroscopy (EDS) detector, dynamic light scattering (DLS; Malvern Zetasizer Nano ZS90, Malvern Instruments Ltd., Worcestershire, UK), UV-Vis spectrophotometer (Hach DR6000), and other physicochemical analyses (pH, electrical conductance). Using DLS and the UV-Vis spectrophotometer, we measured potential stabilities of SNAT and seed NH_2_-AgNPs as a change in hydrodynamic diameter (HDD) and zeta potential as a function of time (0–3 years) at room temperature (25 °C). To decipher SNAT interaction with SARS-CoV-2 and S proteins, HR-TEM (Titan 80-300 TEM, CAMCOR, Oregon, Eugene, OR, USA) coupled with an EDS detector was used (CAMCOR, Oregon, Eugene, OR, USA). Briefly, 50 µL of heat-killed SARS-CoV-2 (isolate USA-WA1/2020; ATCC, Manassas, VA, USA) or recombinant HEK-293-derived SARS-CoV-2 spike (S) protein (R&D Systems Inc., Minneapolis, MN, USA) was combined with 10 µg/mL SNAT (50 µL), gently shaken and incubated at 37 °C for 30 min, and samples were drop casted onto a holey carbon film-coated copper grid and air dried before imaging and EDS elemental mapping occurred. Using the brightfield or STEM mode at 80 kV, images were captured at a magnification of over 200 kx.

### 2.3. Enzyme-Linked Immunosorbent Assay (ELISA)

ELISA was performed in sequence by immobilizing 0.1 µg/mL of multiple recombinant viral proteins, blocking with human BSA, incubating with different concentrations of SNAT, incubating with primary antibodies obtained from bei Resources (Manassas, VA, USA)—including SARS-CoV-2 spike S1 subunit mouse antibody (for S1 subunit), SARS-CoV-2 spike RBD mouse antibody (for RBD) and SARS-CoV-2 spike S2 subunit mouse antibody (for S2 subunit and full-length S protein), and rabbit antibodies KSHV gB52 obtained from Thermo Fisher Scientific (Waltham, MA, USA)—incubating with HRP conjugated appropriate secondary antibodies, and addition of substrate [21]. The absorbance was recorded at 450 nm. Data are presented as means ± SD (error bars) of three experiments.

### 2.4. Infection Assay Conducted Using Calu-3 Cells

Different concentrations of SNAT were mixed with 1 multiplicity of infection (MOI) of SARS-CoV-2 for 30 min at 37 °C before infecting Calu-3 cells for 2 h at 37 °C [17,21]. The toxicity of SNAT against SARS-CoV-2 outside the cell culture would demonstrate its inactivation potential in the form of a virucidal agent with potential to be used as a spray disinfectant on inanimate environments. Unadsorbed virus particles were removed by washing the cells twice with DMEM followed by incubating with infection medium at 37 °C. On the 3rd day post-infection, the supernatant was collected, and virus concentration in the supernatant was determined using RT-qPCR (2019-nCoV RUO kit) as per the manufacturer protocols (Integrated DNA Technologies, Coralville, IA, USA).

### 2.5. SNAT Administration and Experiment

**Figure 1:**
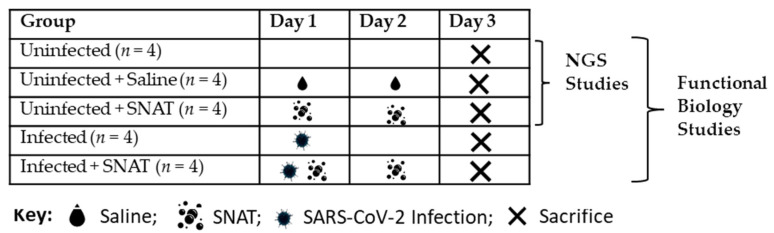
Study design and SNAT dosing: Nebulized using a nose-only inhalation exposure equipment for 20 min at an atomization rate of ≥0.2 mL/min. SARS-CoV-2 dose: Intranasal route (20 µL per nostril) of viable 8 × 10^4^ TCID_50_ virus particles.

We treated each hamster with two doses of 10 µg/mL SNAT (2 mL/dose; 1st dose given 2 h post-infection followed by 2nd dose at 24 h post-infection) in a modified nose-only inhalation exposure equipment [17]. Briefly, the nebulizer (UNOSEK nebulizer) was connected to the modified SCIREQ nose-only exposure chamber that was conveniently fit inside the BSL-3 biosafety cabinet. The nebulizer characteristics are as follows: atomization rate: ≥ 0.2 mL/min; aerosol size range: 0.5–5 µm; mean aerosol diameter: 2.81 ± 0.14 µm; and drug aerosolization rate: 75.7%; capacity: 8 mL max, 0.2 mL/min. Using compressed air technology, the nebulizer allowed for a high nebulization rate. A simplified schematic depicting the study design is presented in Figure 1. The study involved five groups (*n* = 4 per group) of male Syrian hamsters (*Mesocricetus auratus*; Jackson Laboratory) aged 6–8 weeks.

For studies involving next-generation sequencing (NGS), plasma samples from only three groups were used: (i) uninfected; (ii) uninfected + SNAT-treated; and (iii) uninfected + phosphate-buffered saline (PBS)-treated, on day 1 and second dose on day 2, to determine potential effects of SNAT on circulating miRNA expressions in uninfected hamsters. On day 3, the animals were anesthetized via vaporized isoflurane inhalation using an IMPAC6 veterinary anesthesia machine. This study followed ARRIVE guidelines for reporting and all methods were performed according to the American Veterinary Medical Association (AVMA) guidelines.

### 2.6. Preparation of Lung Tissues for Histology and Immunohistochemistry

We collected lung tissues on day 3 post-infection. Tissues were fixed in 10% neutral buffered formalin, trimmed, processed, embedded in paraffin, cut at 5–6 µm, and stained with hematoxylin and eosin (H&E) [17].

Lung sections from SNAT-treated hamsters were processed for immunohistochemistry (IHC). Rabbit primary antibody to IL-6 (1:500 dilution; Abcam, Waltham, MA, USA) was used for immunohistochemical staining. Lung sections were incubated with primary antibodies overnight at 4 °C and developed using ImmPRESS Excel Staining Kit (MP-7601, Vector Laboratories) following manufacturer’s instructions. Using the slide scanner (Philips IntelliSite Ultra-Fast Scanner), the slides were imaged. Representative images were extracted and presented at 40× magnification. Three 40× images were selected from each group and 10 subsections of equal size were taken from each to control for white balance, for a total of 30 subsections used per group for quantification. IL-6 immunoreactivity was assessed from images using ImageJ and presented as optical density, as a proxy to stain intensity. Paired *t*-test was used to test for significant difference between groups at *p* ≤ 0.05.

### 2.7. RT-qPCR to Monitor Expression of Hamster IL-6

Using miRNeasy Serum/Plasma Advanced kit (Qiagen, Germantown, MD, USA), RNA was extracted from the hamster lung specimens. RNA concentrations were measured using a spectrophotometer (NanoDrop ND-2000; Thermo Fisher Scientific, Waltham, MA, USA). Only the RNA samples with 260/280 ratios of 1.8 to 2.0 were used in the study. RT-qPCR was performed using specific primers to IL-6 as per established protocol [22].

### 2.8. Whole Transcriptome Assay

We determined the circulating miRNA profile in the uninfected + SNAT-treated hamster plasma specimens conducting the HTG EdgeSeq miRNA Whole Transcriptome Assay (miRNA WTA) (HTG Molecular Diagnostics, Inc. Tucson, AZ, USA). The HTG EdgeSeq miRNA WTA allows for measuring the expression of 234 hamster miRNA transcripts using next-generation sequencing (NGS). We determined the miRNA expression profile using 20 µL of plasma samples as previously described [22]. We limited the batch effects using the following checklist when performing NGS: samples were randomized in a balanced manner; samples were used in duplicates; correlation of all sample pairs was evaluated; normalization was performed to transform data in such that data from different replicates and experimental conditions were comparable; and a multivariate method of analysis (principal component analysis—PCA) was performed to detect any possible batch effects. PCA was performed based on the Scree plot (eigenvalues vs. components).

### 2.9. Dual-Luciferase Reporter Assay and Western Blotting

The 3′-UTR (with wild-type and mutant binding sites for miR-141-3p) of Ythdf2 (accession number: XM_040756461) and the 3′-UTR (with wild-type and mutant binding sites for miR-200b-3p) of Slit2 (accession number: XM_040747064), respectively, were amplified using PCR and then cloned into XhoI/XbaI site located at 3′-UTR of pmirGLO dual-luciferase vector (Promega, Madison, WI, USA). The 3′-UTR of Ythdf2 and Slit2 with mutations to binding sites (MUT1 and 2) were generated by site-directed mutagenesis as per standard protocols [22]. HEK-293T cells were plated onto 6-well plates. At 24 h post-plating, HEK-293T cells were co-transfected with 3′-UTR luciferase reporter plasmid and miR-mimic (or control mimic) using FuGene HD (Promega). We measured Renilla luciferase activity at 48 h post-transfection using a dual luciferase reporter assay system (Promega) as per manufacturer’s recommendations. The oligos tested in this study are presented in Supplementary Table S1.

We used endotoxin- and pyrogen-free water to make all buffers used in this project. Western blotting was conducted using the following primary antibodies to IL-6 (rabbit polyclonal antibodies; Abcam, Cambridge, UK), Slit2 (rabbit Slit2 polyclonal antibodies; Cell Signaling, Danvers, MA, USA), Ythdf2 (rabbit polyclonal antibodies; Cell Signaling, Danvers, MA, USA), and human β-actin (Cell Signaling, Danvers, MA, USA). The bands were scanned, and the band intensities were assessed using the ImageQuaNT software version 5 (Molecular Dynamics).

## 3. Results

### 3.1. Characterization of SNAT

**Figure 2:**
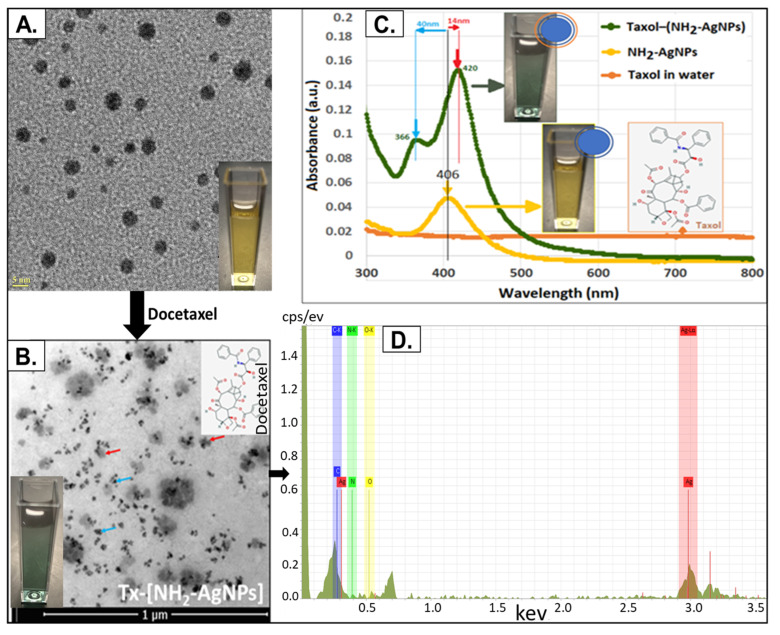
Characterization of SNAT: (**A**) Transmission electron microscopy (TEM) micrographs of amino-functionalized silver nanoparticles (NH_2_-AgNPs) showing spherical particle morphology of elemental/ metallic silver (Ag^0^) with mean particle diameter of 5.8 nm ± 2.8 nm (*n* = 807). Inset photo shows yellow color suspension of NH_2_-AgNPs. (**B**) Electron micrographs of surface Taxol (Tx)-decorated (shown with red arrow) amino-functionalized silver nanoparticles (Tx–[NH_2_-AgNPs]) showing two or more individual NH_2_-AgNPs (shown with blue arrow), each about 5 nm in diameter, self-assembled forming a somewhat triangular nanoparticle architecture that is collectively embedded within Tx molecules. Inset photo shows light blue/green color suspension of Tx–[NH_2_-AgNPs]. (**C**) UV–Vis spectra of docetaxel (Tx)-decorated amino-functionalized silver nanoparticles (Tx–[NH_2_-AgNPs]) (green spectrum/red arrow; (blue) green suspension), NH_2_-AgNPs alone (yellow spectrum, yellow suspension), and Tx alone (in sterile Milli-Q water) (orange spectrum showing a flat line; inset to the right showing molecular structure of Tx). The “seed” of NH_2_-AgNPs alone had a maximum absorbance (λ_max_) at 406 nm, which red-shifted to 420 nm (a change of 14 nm) and blue shifted to 366 nm (a change of 40 nm) upon direct surface binding with Tx molecules. (**D**) Representative EDS spectra of Tx–[NH_2_-AgNPs]. Images are reproduced from our previous publication Ref. [17].

Transmission electron microscopy (TEM) was used to determine the particle size of SNAT. The amino-functionalized silver nanoparticles (NH_2_-AgNPs) had a mean TEM diameter of 5.8 nm ± 2.8 nm (S.D.). SNAT has a spherical morphology with a core composed of an elemental/metallic silver (Ag^0^) surface functionalized with cationic NH_2_ functional group (Figure 2A,B). Surface decorating with docetaxel (Tx) led to the formation of Tx–[NH_2_-AgNPs], i.e., SNAT, whereby two or more individual NH_2_-AgNPs (blue arrow) self-assembled, presenting a near triangular architecture collectively embedded within Tx molecules (red arrow; Figure 2C). The average hydrodynamic diameters (HDDs) for NH_2_-AgNPs and SNAT were similar: 4.3 nm and 5.0 nm, respectively, suggesting that Tx binding did not influence the original TEM particle size of the seed NH_2_-AgNPs. Both NH_2_-AgNPs and SNAT had high positive mean surface zeta potentials: +41 mV and +22 mV, respectively, and were highly stable for over 3 years at room temperature (25 °C) as the standard deviation for HDD was within ±1 nm and within ±3 mV for zeta potential over the 3-year period [17]. The blue-shift of 40 nm and red-shift of 14 nm as demonstrated by the UV–Vis spectra suggest a strong electrostatic binding of anionic Tx with cationic NH_2_-groups on the surface of AgNPs (Figure 2D). The representative EDS spectra demonstrate that the SNAT is composed of Ag, C, O, and N. The dialysis-purified SNAT and NH_2_-AgNP formulations had an alkaline pH (7.5–8.5) and a low electrical conductance of 0.120 mS/cm. We used this SNAT in the entire study to define the mechanism by which SNAT may serve as an antiviral therapeutic against SARS-CoV-2.

### 3.2. SNAT Directly Interacts with SARS-CoV-2

**Figure 3:**
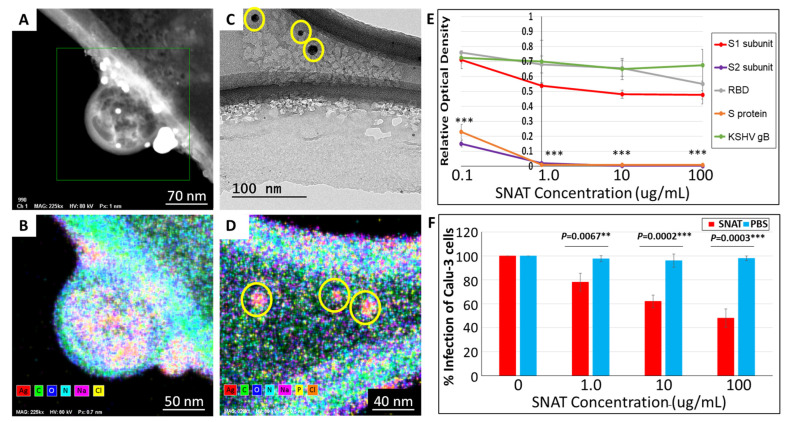
SNAT interactions with SARS-CoV-2. (**A**) HR-TEM analysis of electrostatic interaction between SARS-CoV-2 virus and SNAT particles. (**B**) Energy dispersive spectroscopy (EDS) elemental mapping of SNAT interaction with SARS-CoV-2 virus particle. (**C**) HR-TEM image of SNAT particles physically associated with SARS-CoV-2-encoded S proteins. (**D**) EDS shows elemental/metallic Ag^0^ signature for SNAT interacting with S proteins. (**E**) ELISA of recombinant full-length S protein-His tag, SARS-CoV-2 Spike S1-His Tag, SARS-CoV-2 Spike RBD-His Tag, SARSCoV-2 Spike S2-His Tag protein, and unrelated KSHV gB across varying concentrations SNAT (*** indicates *p* < 0.0005). (**F**) SARS-CoV-2 infection assay using different concentrations of SNAT or PBS treatment. (**B**,**D**) Red denotes silver (Ag), green denotes carbon (C), blue denotes oxygen (O), cyan denotes nitrogen (N), magenta denotes sodium (Na), yellow in panel (**B**) denotes chlorine (Cl), yellow in panel (**D**) denotes phosphorus (P), and brown in panel (**D**) denotes chlorine (Cl). (** indicates *p* < 0.01).

In our previous work, we found that SNAT (10 μg/mL) treatment neutralized SARS-CoV-2 infection in vitro, while the NH_2_-AgNPs (10 μg/mL) or Tx (1 μg/mL) alone could not [17]. Additionally, we showed that SNAT significantly reduces SARS-CoV-2 load in hamsters [17], but the mechanism of action was not deciphered. There are two ways by which an antiviral therapeutic may lower viral infections: (i) by directly binding with the virus and neutralizing infection and (ii) by altering the miRNA-directed cellular milieu to alleviate viral pathogenesis. First, we tested if SNAT could directly bind with the virus employing HR-TEM and EDS. HR-TEM coupled with EDS analyses confirmed that intact SARS-CoV-2 virus electrostatically interacts with several SNAT particles (shown as bright white electron-dense dots in Figure 3A). EDS mapping confirmed the several electron-dense SNAT observed atop and around the virus particles to be composed of elemental Ag^0^ along with C, O, and N (Figure 3B). Significant amounts of C, N, and O signals were also observed for the virus particle (Figure 3B). Interestingly, the HR-TEM images depict SNAT particles physically associated with SARS-CoV-2-encoded S proteins (Figure 3C), while EDS confirmed elemental/metallic Ag^0^ signature for SNAT interacting with S proteins (Figure 3D). However, SNAT did not physically interact with Kaposi sarcoma-associated herpesvirus (KSHV)-encoded glycoprotein B, a functional homologue of SARS-CoV-2-encoded S protein. The results confirm that SNAT specifically interacts with SARS-CoV-2 and the S protein.

To further confirm the ability of SNAT to interact with SARS-CoV-2-encoded S protein, we performed an ELISA. SARS-CoV-2-encoded S protein (1273 aa) comprises an N-terminal signal peptide (1–13 aa), S1 subunit containing the receptor binding domain (RBD (14–685 aa), and S2 subunit (686–1273 aa) [22,23]. The rationale for this experiment was that if SNAT is bound to the S protein, it may hinder the ability of antibodies to bind to the protein. We tested recombinant full-length S protein-His tag, SARS-CoV-2 Spike S1-His Tag, SARS-CoV-2 Spike RBD-His Tag, SARSCoV-2 Spike S2-His Tag protein, and unrelated KSHV gB [24] in this assay (Figure 3E). Our results demonstrated the ability of SNAT to physically interact with the full-length S protein and the S2 subunit contained within it compared to the S1 subunit and RBD of the S protein, BSA, or KSHV gB (Figure 3E). If this is true, SARS-CoV-2 infection of cells in the presence of SNAT must be significantly lowered. Hence, we tested the ability of different concentrations of SNAT to inhibit SARS-CoV-2 infection of Calu-3 cells. Interestingly, SNAT at ≥1 µg/mL could significantly lower SARS-CoV-2 infection in vitro (Figure 3F). We observed a maximum of 55 ± 7.6% drop in viral infectivity in the presence of SNAT when compared to the vehicle (PBS) or untreated cells. Taken together, our results indicate that SNAT can neutralize viral infection by physically interacting with the S2 subunit of SARS-CoV-2-encoded S protein.

### 3.3. SNAT-Induced Differential Expression of Circulatory miRNAs in Uninfected Hamsters

**Figure 4:**
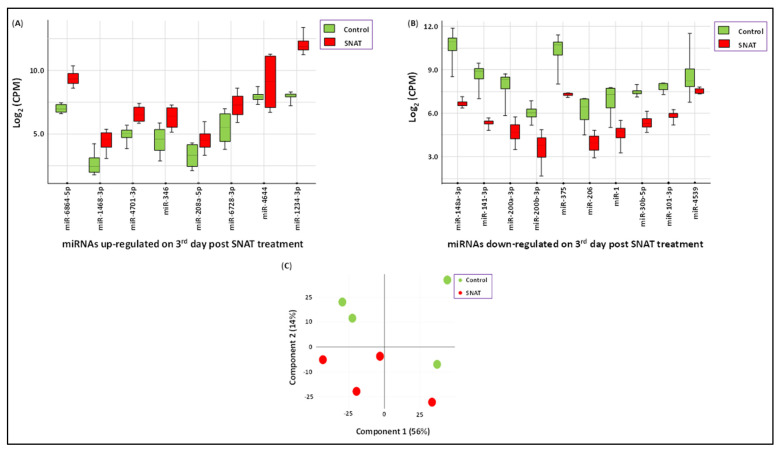
SNAT-induced differential expression of circulatory miRNAs in uninfected hamsters. (**A**) Upregulated and (**B**) downregulated expression of miRNAs in untreated, uninfected hamsters (green; control) compared to uninfected hamsters treated with SNAT (red). (**C**) PCA plot showing different clustering of the two groups.

**Figure 5:**
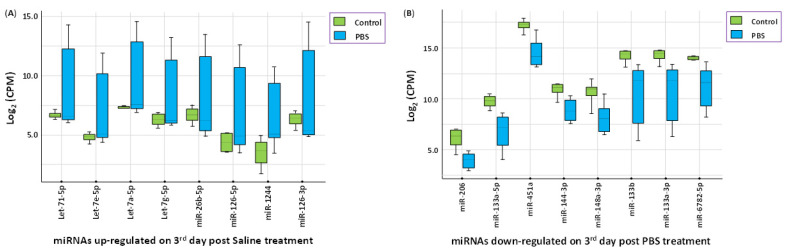
PBS treatment in hamsters did not significantly alter the circulatory micronome. (**A**) Upregulated and (**B**) downregulated expression of miRNAs in untreated, uninfected hamsters (green) compared to uninfected hamsters treated with phosphate-buffered saline (PBS; blue). PCA plot is presented in Supplementary Figure S1A.

We assessed the potential direct effects of SNAT treatment on hamsters by screening for changes in the circulatory micronome using the extraction-free whole transcriptome assay. We observed intranasal SNAT treatment to significantly alter 18 miRNAs in uninfected hamsters on day 3 post-treatment. A total of 8 miRNAs were up-regulated, while 10 miRNAs were down-regulated by SNAT treatment when compared to untreated and uninfected (control) hamsters (Figure 4A,B). Principal component analysis (PCA) showed that the miRNA expression profiles in SNAT-treated hamsters and untreated hamsters were segregated into distinct clusters along principal component 1 and principal component 2, explaining 56% and 14% of the total variances (Figure 4C). Administration of PBS to uninfected hamsters did not significantly alter the circulatory micronome when compared to those that were untreated and uninfected (Figure 5). The PCA plot showed no separation between the two groups (PBS treated and untreated), meaning there was no distinct clustering of the points for the two groups, and the points from the two groups appeared to be evenly intermixed. The scree plots are provided in Supplementary Figure S1, depicting principal components and their eigenvalues.

### 3.4. SNAT Promotes Recovery from SARS-CoV-2 Viral Infection by Altering Select Circulatory miRNAs in Hamsters

**Figure 6:**
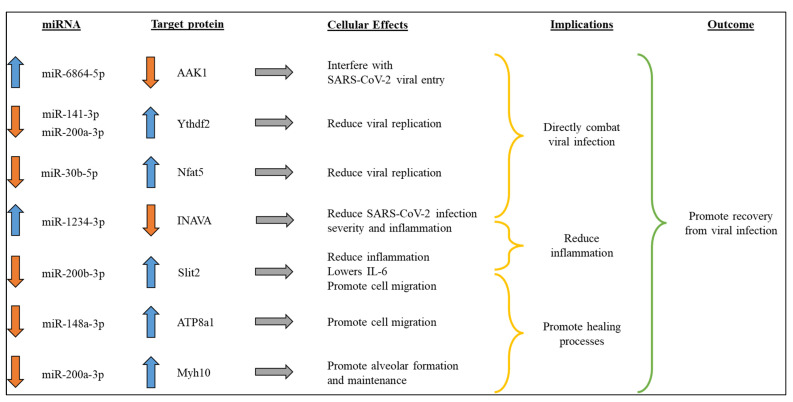
Differentially expressed miRNAs in hamsters treated with SNAT and potential effects of altered expression of physiologically relevant targets.

Of the multiple circulatory miRNA expressions that were altered, we focused on the seven most physiologically relevant miRNAs, as depicted in Figure 6. Upregulation of miR-6864-5p and miR-1234-3p along with the downregulation of miR-141-3p, miR-200a-3p, and miR-30b-5p could potentially lower SARS-CoV-2 replication [25,26,27,28]. Enhanced miR-1234-3p expression accompanied by a decrease in miR-200b-3p can potentially reduce inflammation [28,29,30,31]. A significant decline in the expression of miR-148a-3p and miR-200a-3p may promote healing processes [32,33,34,35,36]. The overall result of these changes to the miRNA expression profile observed in SNAT-treated hamsters is decreased viral load and inflammation, while promoting the healing process, all of which support recovery from SARS-CoV-2 infection.

The rationale for probing for the expression of IL-6 (downstream target of miR-200b-3p), Ythdf2 (target of miR-141-3p), and Slit2 (target of miR-200b-3p) in SNAT-treated hamster lungs are as follows: (i) SARS-CoV-2 infection is known to increase expression of IL-6 in hamsters as well as in humans [37] and (ii) our luciferase reporter assay confirmed Ythdf2 and Slit2 to be direct targets of miR-141-3p and miR-200b-3p, respectively (Supplementary Figure S2). The active miR-141-3p and miR-200b binding sites in 3′-UTR of Ythdf2 and Slit2 mRNA, respectively, are provided in Supplementary Figure S4.

**Figure 7:**
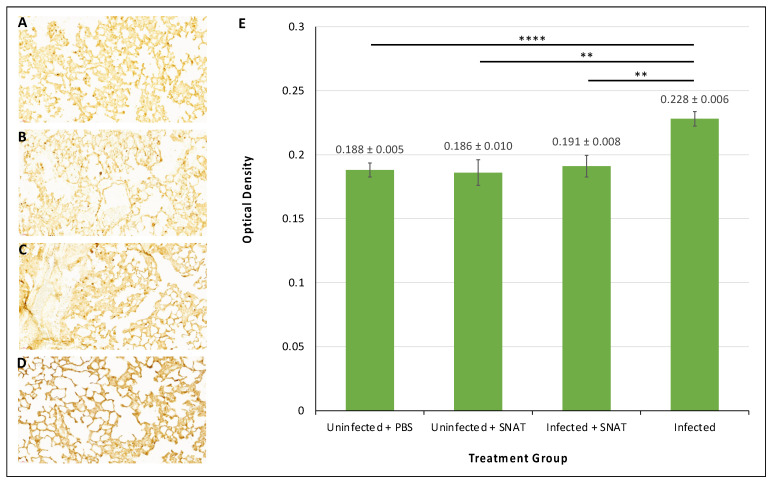
Immunohistochemical analysis of IL-6 expression in hamster lung. 3,3′-Diaminobenzidine (DAB) stain for IL-6 in uninfected hamsters treated with PBS (**A**) or SNAT (**B**) and hamsters infected with SARS-CoV-2 treated with SNAT (**C**) or untreated (**D**) at 40× magnification. (**E**) IHC quantification of IL-6 for each group using optical density with standard error bars. Significance determined by *p* value < 0.05 using two-tailed paired *t*-test (** indicates *p* < 0.01; **** indicates *p* < 0.0001; no asterisk indicates no significant difference between groups).

The immunohistochemistry results indicated no significant difference in IL-6 expression in the lungs that were uninfected and treated with PBS or uninfected and treated with SNAT (Figure 7A,B). SNAT did not seem to have any significant adverse effects on the lung tissues when analyzed by H&E staining (Supplementary Figure S3). We also tested IL-6 expression in lungs derived from SARS-CoV-2-infected hamsters and SARS-CoV-2-infected hamsters treated with SNAT, 3 days post-infection. The IL-6 expression in lungs from infected hamsters was significantly elevated (Figure 7D) compared to uninfected hamsters treated with PBS or uninfected hamsters treated with SNAT (Figure 7A,B). Also, SNAT-treated SARS-CoV-2-infected hamsters expressed significantly lower levels of IL-6 (Figure 7C) compared to SARS-CoV-2-infected hamsters that did not receive this treatment (Figure 7D). Raw data corresponding to relative IL-6 levels are provided for comparisons (Figure 7E).

**Figure 8:**
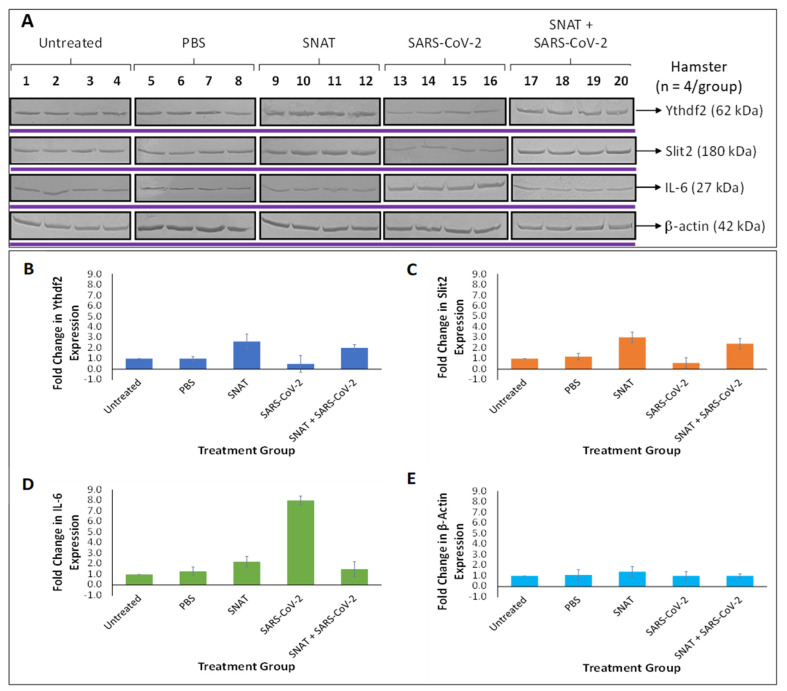
Western blot results (**A**) and graphical representation of protein expression of (**B**) Ythdf2, (**C**) Slit2, (**D**) IL-6, and (**E**) β-actin in uninfected, untreated hamsters (*n* = 4), treated with PBS (*n* = 4), treated with SNAT (*n* = 4), untreated, infected hamsters (*n* = 4), or infected hamsters treated with SNAT. β-actin was used as a control and showed no significant difference in expression between groups. The gels from the same blot are boxed in black lines and the blots for different antibodies are demarcated with purple lines. High-contrast blots were not used to generate this figure. The graphical representation of the respective protein bands was generated by measuring the Western blot band intensities using the ImageQuaNT software version 5 (Molecular Dynamics).

To further confirm the effects of SNAT on hamster lungs, we performed Western blotting using lysates derived from the lung specimens below. Our data demonstrate the ability of SNAT to significantly lower IL-6 expression compared to hamsters that were infected with SARS-CoV-2, while increasing the expression of Ythdf2 and Slit2 when compared to uninfected controls (Figure 8). These data confirm our model proposed in Figure 6.

## 4. Discussion

SNAT was previously shown to combat SARS-CoV-2 infection by lowering viral titer, improving lung health, and reversing virus-induced weight loss in golden Syrian hamsters [17]. Here, we elucidated potential mechanisms underlying SNAT inhibition of SARS-CoV-2 infection in hamsters. We determined two potential explanations for its antiviral properties: SNAT could be directly interacting with the virus to prevent infection of cells and/or could be affecting the global transcriptome of the host to indirectly interfere with viral pathogenesis.

High-resolution TEM imaging revealed that SNAT directly binds to SARS-CoV-2 to block SARS-CoV-2 infection (Figure 3). To investigate how this occurs, we focused our efforts on S protein because (i) S protein is a glycosylated protein that is expressed on the viral envelope [38] and (ii) S protein plays a crucial role in the virus entry process [39]. We did not focus on the M protein that is also expressed on the viral envelope because it plays a role in the viral assembly and not in the entry process [40,41]. Targeting the S protein with therapeutics may prevent viral entry and reduce infectivity. The S protein comprises two subunits, with S1 predicted to house the receptor binding domain (RBD) and S2 predicted to play a role in viral fusion [42].

Our ELISAs confirmed that SNAT selectively binds to the S2 subunit contained within the S protein (Figure 3E). Though the SARS-CoV-2 virus has an overall positive charge, the S protein is net negative, with 99 cationic and 111 anionic amino acid residues [43]. The S2 subunit has a net negative charge, with 14 positive and 18 negative amino acids [43]. This suggests that electrostatic interactions with the positively charged SNAT particles may occur. In contrast, the S1 subunit of the S protein is positive, with 7 cationic amino acid residues in the RBD resulting in electrostatic attractions with the 28 anionic residues in the ACE2 receptor [43]. We hypothesize that the excess negative charge at the S2 subunit of S protein is crucial to the binding of SNAT, which interferes with viral entry at the receptor interface.

Disulfide bonds between the S protein and ACE2 receptor have been implicated in viral binding and AgNPs have been shown to disrupt disulfide bonds [18]. Furthermore, AgNPs preferentially bind to viral surface proteins rich in sulfhydryl groups [18]. The S proteins of SARS-CoV-2 variants contain 30 conserved cysteine residues on the ectodomain that form specific disulfide bonds within the protein [44]. This suggests that SNAT may disrupt disulfide bridges between the S protein and ACE2 to impede viral binding, as well as impair the structural stability of the S protein across multiple variants of SARS-CoV-2.

SNAT can also affect the global transcriptome of the host by altering miRNA levels (Figure 4 and Figure 5). Therefore, we chose to examine the effects of SNAT alone on the cellular milieu in vivo. We only used SNAT and its vehicle PBS in this assay as there is no report on the effects of SNAT or similar therapeutics on the micronome in hamsters and mice models. We deliberately avoided the use of SARS-CoV-2 as there are multiple papers to show how the virus alters miRNA profiles in various rodent models, including hamsters [44,45]. Our extraction-free whole transcriptome assay revealed significant differences in the expression levels of eight physiologically relevant circulatory miRNAs (Figure 6). Using miRBD and miRBase, we investigated potential targets of these miRNAs and their cellular functions. These results informed our predictive model (Figure 5) that supports the ability of SNAT to promote recovery from viral infection by reducing inflammation, promoting healing processes, and inhibiting viral replication.

Western blotting experiments were performed to verify the effects of SNAT treatment on protein expression in hamsters. Our results indicated that SNAT-induced upregulation of miR-141-3p decreased the expression of target protein Ythdf2 (Figure 8). Decreased Ythdf2 has been implicated in increased lentiviral protein expression and production of lentiviruses in vitro [34]. Additionally, Ythdf2 is involved in the negative regulation of the innate immune response by RNA degradation of IKKγ and p65 transcripts, and silencing of Ythdf2 resulted in reduced viral replication of vesicular stomatitis virus (VSV) in vivo [26]. Since the innate immune response is non-specific, it is plausible that downregulation of Ythdf2 could increase cellular expression of these antiviral transcripts and bolster the innate immune response during SARS-CoV-2 infection.

Our results suggested that SNAT-induced downregulation of miR-200b-3p increased the expression of target protein Slit2, which is significantly downregulated by SARS-CoV-2 infection (Figure 8). Slit2 is involved in inflammatory processes and has been demonstrated to reduce IL-6 levels via the NF-κB pathway [46]. This may help explain lower IL-6 expression in lungs derived from infected hamsters treated with SNAT compared to untreated, infected hamsters (Figure 7). In addition, decreased inflammation may contribute to reduced lung injury as observed in SARS-CoV-2-infected hamsters treated with SNAT compared to infected hamsters without treatment [17].

Nanomedicines such as SNAT have many advantages over traditional antivirals. The method of drug administration or engineered coatings on the surface of the NP may enable targeting of specific cell types or tissues [47]. SNAT was delivered intranasally, allowing for more direct drug delivery than intravenous or oral alternatives to the lungs, an area of concern in respiratory illnesses such as SARS-CoV-2 infection [17,48]. The size of the nanoparticle can also affect delivery. SNAT nanoparticles have a hydrodynamic diameter (HDD) of 5.0 nm, on average, within the size range of inhalable particles that localize to the tracheobronchial and alveolar regions of the human respiratory tract [17,47].

In addition, spherical nanoparticles such as SNAT have more effective cellular uptake than non-spherical nanomaterials (rods, cylinders, and cubes) under 100 nm [17,49]. Nanodrugs can also have a longer half-life, which, in combination with the size and targeted delivery, may reduce dosages [11]. In turn, this could result in reduced adverse effects and increased patient compliance compared to traditional regimens [11]. As little as only two doses of 10 µg/mL SNAT (2 mL/dose, given 24 h apart) could demonstrate potent antiviral effects in hamsters infected with SARS-CoV-2 [17]. Furthermore, the documented toxicity of SNAT against SARS-CoV-2 outside the cell culture suggests its potential as a virucidal agent that may find applications as a spray disinfectant on inanimate surfaces and environments.

Other AgNPs have been used against SARS-CoV-2; however, few in clinical trials focus on severe infection and preventative measures. For example, Wieler et al. used AgNPs as an adjuvant treatment for SARS-CoV-2-infected patients (*n* = 40) with virus-induced pneumonia and demonstrated a significant improvement in survival rate [50]. Yet, with intravenous administration across three consecutive days, this treatment requires consistent patient access to a healthcare facility [50]. This regimen is well suited for hospitalized patients with moderate to severe infections; however, it may not be conducive to outpatient treatment. In contrast, SNAT treatments can be administered at home with ease via a nebulizer [17].

Another study by Almanza-Reyes et al. utilized AgNPs in a nose rinse and mouthwash to prevent SARS-CoV-2 infection among high-risk healthcare workers [51]. Treatment significantly reduced the incidence of infection with only 2 out of 114 participants in the experimental group, compared to 33 out of 117 in the control group, testing positive over 9 weeks [51]. This method of administration targets the nasal and oral cavities, which are areas of entry for the virus [48,51]. In contrast, SNAT targets the airways and penetrates into the lungs, making it better suited for patients with active infections. SNAT has not yet been studied in the context of prevention; however, this may be an interesting direction for future works.

The multitarget mechanisms of action exhibited by AgNPs and the potential for surface modifications with desired small molecules/ligands make them a promising candidate for mitigating viral infections, antiviral resistance and managing comorbidities often associated with complex viral infections like COVID-19. Given its multitarget-directed actions, there is increased research interest in exploring the preventative and therapeutic efficacy of AgNPs against various viral diseases affecting public heath [52,53]. Apart from AgNPs, studies have explored antiviral and/or virucidal properties of ceria nanoparticles. For example, Zhang et al. reported higher anti-SARS-CoV-2 efficacy in vitro of 3 nm cerium oxide nanoparticles (CeO_2_NP-3 nm) compared to 30 nm CeO_2_NP (CeO_2_NP-30 nm) [54]. The authors documented that the CeO_2_NP-3 nm targeted the 5 nm S protein trimer cavity, enabling binding with the RBD, thereby blocking the virus-ACE2 interaction and inhibiting viral cell entry [54]. However, in vivo effects were not studied. Other studies have implicated the release of cerium ions, generation of reactive oxygen species, and/or acting as a DNA cutter on the antiviral/virucidal mechanisms of action of ceria-based nanoparticles [55,56].

Overall, our findings suggest that SNAT may serve as a safer antiviral and virucidal agent against SARS-CoV-2. While Wieler et al. did not define the exact mechanism of their AgNPs, they hypothesized that they may interfere with viral binding and trafficking, binding to the viral genome to combat infection, and/or reducing inflammatory cytokines to prevent cytokine storms [50]. We have gone further to illuminate the mechanism of SNAT and its direct and indirect effects on viral infection, which may help inform future clinical studies. Both Wieler et al. and Almanza-Reyes et al. reported no adverse events from the use of their AgNPs [50,51], a result consistent with our findings.

## 5. Conclusions

Herein, we propose molecular mechanisms underpinning SNAT inhibition of SARS-CoV-2 infection and ensuing pathogenesis in a hamster model. By binding to the S2 subunit of the S protein of SARS-CoV-2, SNAT interferes with viral fusion to prevent infection of cells. It may also disrupt the stability of the S protein and impair viral binding to ACE2 receptors to prevent viral entry. In addition, SNAT treatment alters the cellular environment through transcriptomic changes to help neutralize infection. It downregulates miR-200b-3p to increase the expression of Slit2, lowering inflammation, including IL-6 expression. Furthermore, it upregulates miR-141-3p to decrease the expression of Ythdf2, which reduces viral replication and promotes the innate immune response. The antiviral effects. as observed on the miRNA-directed milieu. indicate broader applications of SNAT as medical countermeasures against viral respiratory infections. In addition, SNAT may also serve as a virucidal agent to disinfect inanimate surfaces and environments. As research continues to progress in this field, nanomedicines such as SNAT may hold promise for combating current and future viral outbreaks.

## 6. Patents

SNAT was synthesized by L.R.P. using the “seed” of NH_2_-AgNPs (International Patent Application No. PCT/US2020/014343; US Patent Application No. 17/759,260; European Regional Phase Application No. 21744759.8).

## Data Availability

The data supporting the findings of this study are available from the corresponding author(s) upon reasonable request.

## Supplementary Materials

The following supporting information can be downloaded at: https://www.mdpi.com/article/10.3390/toxics12070495/s1, Supplementary Figure S1: PCA plot showing similar clustering of the uninfected + PBS-treated (blue) and uninfected + untreated groups (green). Figure S2: miR-200b-3p and miR-141-3p specifically binds and interact with Slit2 and Ythdf2, respectively. Figure S3: Hematoxylin and eosin (H&E) staining of the lungs of uninfected hamsters (1) control, (2) with SNAT treatment, or (3) with saline treatment at (a) 40× and (b) 200× magnification. Figure S4: RNA hybrid analysis shows miR-141-3p and miR-200b binding site in 3’-UTR of Ythdf2 and Slit2 mRNA. This is predicted using miRDB and TargetScan algorithms and Supplementary Table S1: Oligos used in the Luciferase assay.
