# Supplementary material for: Evaluating Molecular Mechanism of Viral Inhibition of Aerosolized Smart Nano-Enabled Antiviral Therapeutic (SNAT) on SARS-CoV-2-Infected Hamsters
Source: Toxics. 2024 Jul 5;12(7):495. doi: 10.3390/toxics12070495 (PMC11280845; doi:10.3390/toxics12070495)
Supplement: Supplementary file 1 [file toxics-12-00495-s001.zip › toxics-3050893-supplementary.pdf]

Evaluating molecular mechanism of viral inhibition of aerosolized Smart Nano-enabled Antiviral Therapeutic (SNAT) on SARS-CoV-2 infected hamsters

Anais N. Bauer[1], John F. Williams[1], Lok R. Pokhrel[2]*, Selena Garcia[1], Niska Majumdar[1], Jeffrey B. Eells[3], Paul P. Cook[4], Shaw M. Akula[1,4]*

[1] Department of Microbiology and Immunology; [2]Department of Public Health; [3]Department of Anatomy and Cell Biology; [4]Department of Internal Medicine, Brody School of Medicine at East Carolina University, Greenville, NC 27834.

***Corresponding author:**
*Shaw M. Akula - Department of Microbiology & Immunology, Brody School of Medicine at East Carolina University, Greenville, NC 27834. Tel: +1 252 744 2702; Fax: +1 252 744-3104; Email: akulas@ecu.edu

*Lok R. Pokhrel - Department of Public Health, Brody School of Medicine at East Carolina University, Greenville, NC 27834. Tel: +1 252 744 3775; Fax: +1 252 744-4272; Email: pokhrell18@ecu.edu

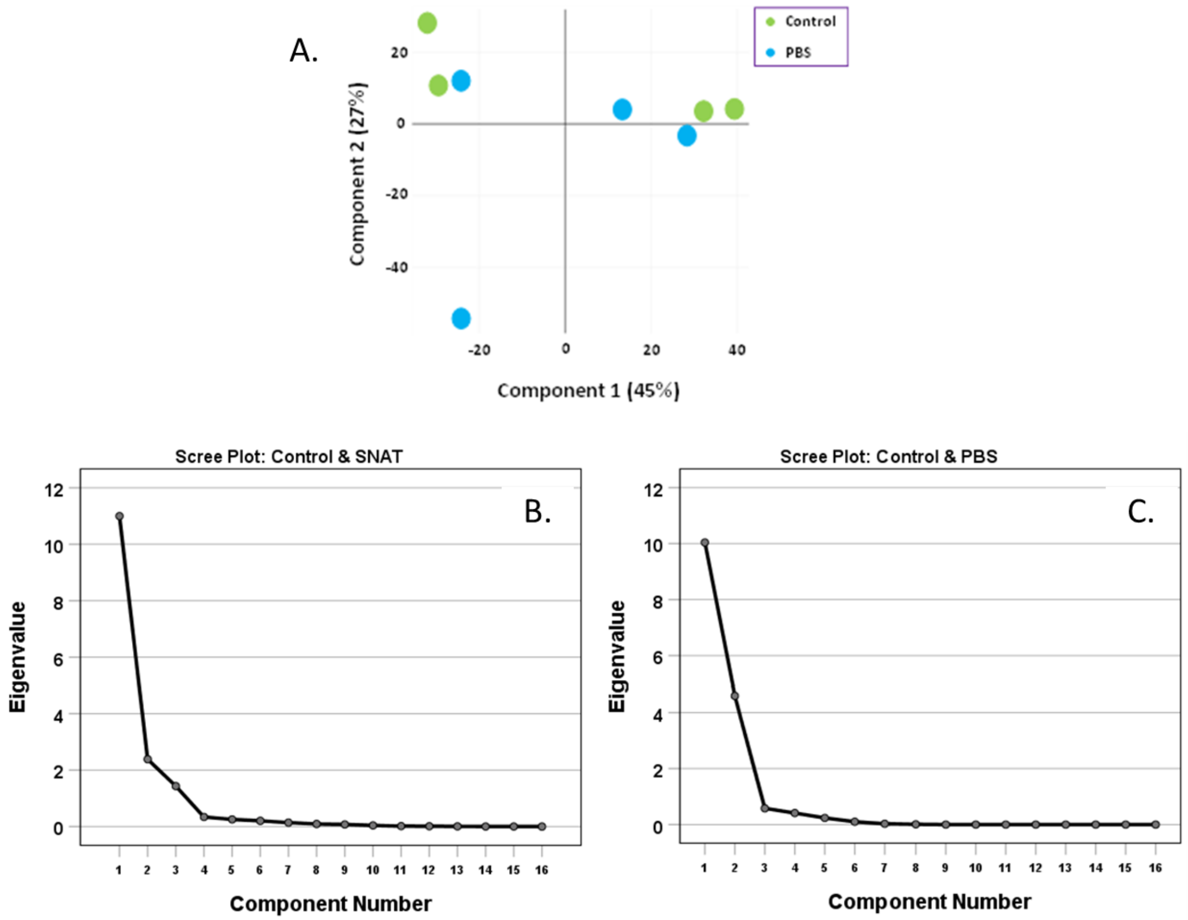

Supplementary Figure S1. PCA plot showing similar clustering of the uninfected + PBS-treated (blue) and uninfected + untreated groups (green) (details on circulatory miRNAs are presented in main text Fig. 4). Scree plots (**B, C**) showing eigenvalues for the principal components presented in Figures 3 (in the main text) and S1A (above). (**B, C**) Scree plots showing eigenvalues on the Y-axis and the number of factors on the X-axis for transcriptome differences between miRNA expression in uninfected and untreated Control, uninfected and SNAT treated, and uninfected and PBS-treated groups of golden Syrian hamster lungs.

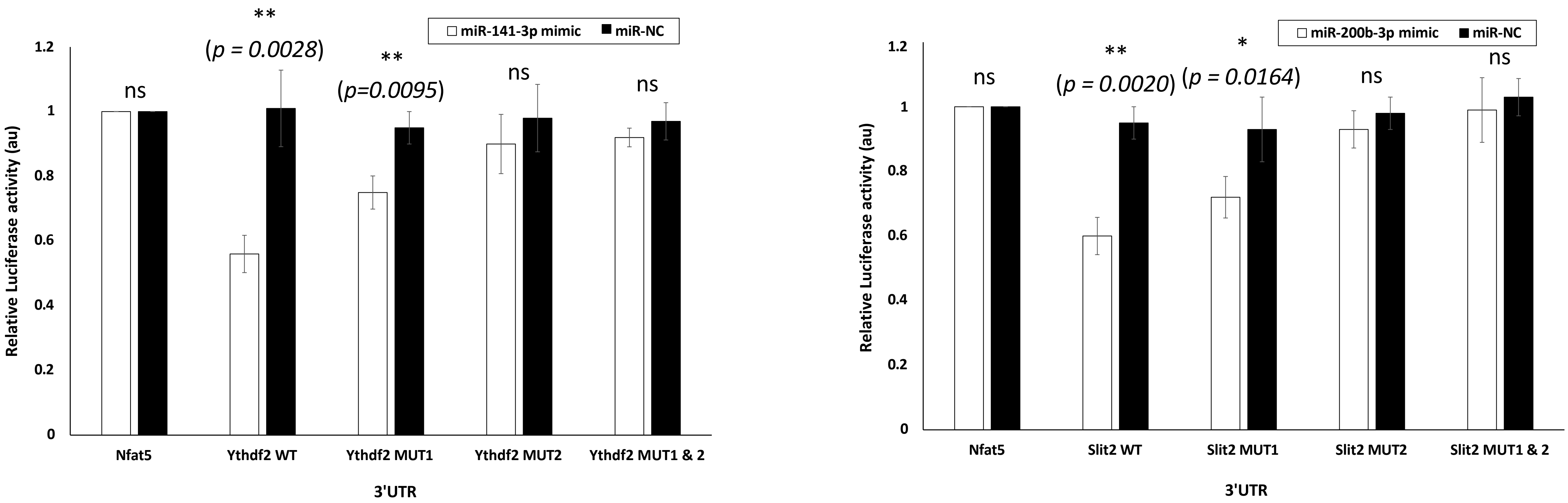

Supplemental Figure S2: miR-200b-3p and miR-141-3p specifically binds and interact with Slit2 and Ythdf2, respectively. HEK293T cells were transfected with (i) Slit2 3'UTR, Ythdf2 3'UTR, or Nfat5 3'UTR (*Control*); (ii) co-transfected with vector encoding the above Slit2 3'UTR and miR-200b-3p mimic, co-transfected with Slit2 3'UTR and control mimic (miR-NC); (iii) co-transfected with vector encoding the above Ythdf2 3'UTR and miR-141-3p mimic, co-transfected with Ythdf2 3'UTR and control mimic (miR-NC). We also tested Slit2 and Ythdf2 MUT1 and MUT2 3'UTR in this study. Luciferase activity was monitored at 48 h post-transfection and was normalized against the effect of the miRNAs on the Firefly luciferase reporter alone. The relative luciferase activity for cells transfected with Slit2 3'UTR, Ythdf2 3'UTR, or Nfat5 3'UTR is considered as 1 au. The x-axis denotes the UTRs transfected, and y-axis indicates the relative luciferase activity. Bars represent average ± s.d. of three individual experiments. One-way ANOVA was performed compare between multiple groups. The p value and their significance is indicated; 'ns' denotes not significant at p=0.05.

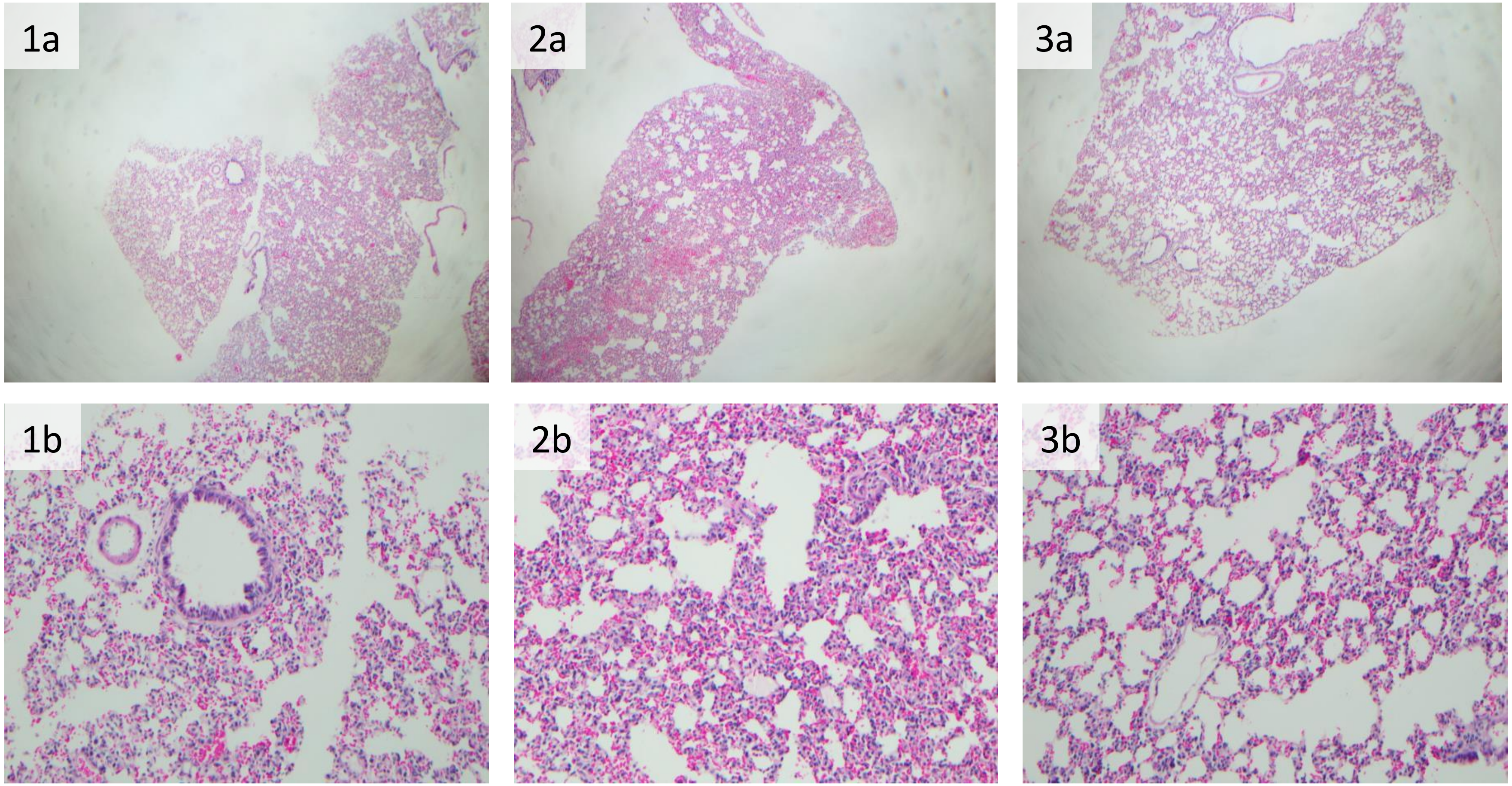

Supplementary Figure S3: Hematoxylin and eosin (H&E) staining of the lungs of uninfected hamsters (1) control, (2) with SNAT treatment, or (3) with saline treatment at (a) 40X and (b) 200X magnification.

Supplementary TABLE S1: Oligos used in the Luciferase assay.

**Name**	**Sequence**
miR-141-3p mimic	UAACACUGUCUGGUAAAGAUGG
miR-200b-3p mimic	UAAUACUGCCUGGUAAUGAUGA
miR-NC	ucacaaccuccuagaaagaguaga
WT hamster Ythdf2 3'UTR sequence	CTCGAGtttttgtctaggagagtttaataa**cagtgtta**
Hamster Ythdf2 3'UTR sequence Mutant-1	CTCGAGt**acccaacc**aggagagtttaataa**cagtgtta**
Hamster Ythdf2 3'UTR sequence Mutant-2	CTCGAGtttttgtctaggagagtttaataa**acccaacc**
Hamster Ythdf2 3'UTR sequence Mutant-1&2	CTCGAGt**acccaacc**aggagagtttaataa**acccaacc**
WT hamster Slit2 3'UTR sequence	CTCGAGcgaaggtgtttatctaccactttc**cagtatta**
Hamster Slit2 3'UTR sequence Mutant-1	CTCGAGc**acccaacc**ttatctaccactttc**cagtatta**
Hamster Slit2 3'UTR sequence Mutant-2	CTCGAGcgaaggtgtttatctaccactttc**acccaacc**
Hamster Slit2 3'UTR sequence Mutant-1&2	CTCGAGc**acccaacc**ttatctaccactttc**acccaacc**

miRNA binding sequence (red)

Mutated sequence (green)

miR-141-3p : Ythdf2 interactions
Hamster Ythdf2: XM_040756461

Position 2573-2582 of Ythdf2 3'UTR	5'	G G A G A G T T T A A T A A C A G T G T T A	3'
miR-141-3p	3'	G G U A G A A A U G G U C U G U C A C A A U	5'

miR-200b-3p : Slit2 interactions
Hamster Slit2: XM_040747064

Position 7075-7082 of Slit2 3'UTR	5'	T A T C T A C C A C T T T C C A G T A T T A	3'
miR-200b-3p	3'	A G U A G U A A U G G U C C G U C A U A A U	5'

Supplementary Figure S4. RNA hybrid analysis shows miR-141-3p and miR-200b binding site in 3'-UTR of Ythdf2 and Slit2 mRNA. This is predicted using miRDB and TargetScan algorithms.
